# Development of a framework for the implementation of electronic interventions in mental health: a protocol for a meta-synthesis of systematic reviews

**DOI:** 10.12688/f1000research.27150.1

**Published:** 2020-10-29

**Authors:** David Villarreal-Zegarra, Christoper A. Alarcon-Ruiz, G.J. Melendez-Torres, Roberto Torres-Puente, Juan Ambrosio-Melgarejo, Alejandra B. Romero-Cabrera, Ana Lindo-Cavero, Jeff Huarcaya-Victoria

**Affiliations:** 1 Instituto Peruano de Orientación Psicológica, Lima, Peru; 2 CRONICAS Center of Excellence in Chronic Diseases, Universidad Peruana Cayetano Heredia, Lima, Peru; 3 Instituto de Investigación, Universidad Católica Los Ángeles de Chimbote, Lima, Peru; 4 Universidad Científica del Sur, Lima, Peru; 5 Peninsula Technology Assessment Group, College of Medicine and Health, University of Exeter, Devon, UK; 6 Departamento de Psiquiatría, Hospital Nacional Guillermo Almenara Irigoyen, Lima, Peru; 7 Centro de Investigación en Salud Pública, Facultad de Medicina, Universidad de San Martín de Porres, Lima, Peru

**Keywords:** Telemedicine, Remote Consultation, Online Systems, Internet-Based Intervention, Mental Health, Mental Disorders, Systematic Reviews as Topic, Qualitative Research

## Abstract

**Background: **During the COVID-19 pandemic, it has been necessary to incorporate technologies in the care of mental health problems. But there have been difficulties in the application of technology-based interventions in mental health. Some quantitative systematic reviews don’t allow us to fully identify and properly describe this subject. In order to answer the question "how do electronic interventions apply in mental health and what makes the application of any of these interventions work", this study will carry out an overview of systematic reviews, which will make it possible to develop a theoretical framework on the implementation of electronic care in mental health problems.

**Methods: **We will search MEDLINE, EBM Reviews, PsycINFO, EMBASE, SCOPUS, CINAHL Complete, and Web of Science databases from 1st January 2015 to September 2020, with no language restriction. We will follow a qualitative method approach and include systematic reviews that assess primary studies relating to adults with common mental health problems using any type of mobile mental health intervention that includes a synchronic component and communication with a mental health professional. For the analysis, we will make a meta-synthesis of the systematic reviews, using an emergent grounded theory approach to synthesize the information, prioritizing the systematic reviews with the lowest risk of bias in the AMSTAR-2 tool. The meta-synthesis will be based on interpreting, integrating, and inferring the evaluation elements to understand better the e-health implementation process for patients with mental health problems. Finally, we will present the overall assessment in a Summary of Qualitative Findings table.

**Conclusion: **Our results will allow a better understanding of the facilitator and limitations in implementing e-health interventions for mental health problems.

## Introduction

In the wake of the COVID-19 pandemic and after the actions taken by governments (such as social isolation), many mental health problems have increased in patients with COVID-19, patients with psychiatric symptoms, health personnel, and the general population ^1, 2^. As a result, greater interest has been taken in addressing mental health issues during the pandemic ^2, 3^. An example of this are the studies that identify anxiety disorders, depression, post-traumatic stress disorder, and stress as the most frequent health problems ^2– 4^. To deal with these mental health problems and considering the current context (remote attention), it has been necessary to incorporate the use of technologies in the care ^5, 6^ These technologies have been very well received and have served to complement or improve the effectiveness of treatments for various chronic diseases, reducing gaps in access to care and providing specialized care in inaccessible places ^7^. In addition, these interventions using technologies show great promise in the care of mental health problems ^8– 10^, highlighting the possibilities of care using technologies in health systems where resources are limited ^11^.

With the undeniable contribution of the use of technologies in mental health care, it has been important to document the aspects related to the application of interventions using technologies, since despite the effectiveness that these interventions can have, it is known that there have been difficulties in the application of interventions using technology ^12^. Aspects such as adaptability, cost, complexity, external policies and incentives, compatibility or general fit between the e-health intervention and the organization, etc. ^13^ are important to consider in order to be clear about how and what works in these interventions and consider its complexity. To try to answer this there are many studies which have gathered information and synthesized it. An example of this are the systematic reviews and meta-analyses of interventions in mental health care using technologies (the latter also allowed conclusions to be drawn about the effectiveness of the interventions used) ^14, 15^. However, there are certain aspects which this type of review (centered on quantitative studies) does not allow to identify ^16^. That is why in order to answer the question "how do electronic interventions apply in mental health and what makes the application of any of these interventions work"; this study will carry out an overview of systematic reviews, which will make it possible to develop a theoretical framework on the implementation of electronic care in mental health problems.

## Methods

### SPIDER question

This protocol adheres to the Preferred Reporting Items for Systematic Reviews and Meta-Analyses Protocols (PRISMA-P) guidelines ^17^ A completed PRISMA-P checklist can be found in the *Reporting guidelines* section ^18^. We used the SPIDER framework to develop the review question ^16^:

- *Sample:* Adults with depression (or major depressive disorder), anxiety (or generalized anxiety disorder), stress (or trauma-related disorders), and/or general mental health problems (unspecified).
- *Phenomenon of interest:* Any type of mobile mental health intervention that includes a synchronic component, communication with a mental health professional (psychiatrist, psychologist, etc.) or a health professional trained in mental health. These interventions include, among others, remote consultation, interactive application, video chats, calls, etc.
- *Design:* Systematic review.
- *Evaluation:* We will include all types of outcomes of interest assessed by implementation studies, economic, qualitative, quantitative and others. For example, a) Health effectiveness outcomes: Depression, anxiety and/or stress symptoms, adherence to treatment, etc; b) Patient outcomes: Quality of life, satisfaction, etc; c) Economic outcomes; d) Damage or adverse effects. Also, we will include all kinds of statistical measures of the effects, if available: Relative risks, odds ratio, risk difference, mean difference and/or number needed to treat.
- *Research type:* Quantitative studies, qualitative studies, mixed methods.

### Eligibility criteria

*Inclusion criteria:* Systematic reviews that report on inclusion/exclusion criteria, conducted an adequate search, synthesized the included studies, assessed the quality of the included studies, and presented sufficient detail on the individual included studies ^19^. Systematic reviews that included primary studies as a unit of analysis focused on a research question. Systematic reviews that were published in the last five years (since January 1, 2015) without language restrictions. We are including this time frame in order to include only the last updated systematic reviews. Reviews must include primary studies relating to adults with common mental health problems: a) Adults with depression (or major depressive disorder), anxiety (or generalized anxiety disorder), stress (or trauma-related disorders) and/or general mental health problems (unspecified); b) Adults attending an outpatient mental health consultation.

*Exclusion criteria:* Narrative reviews, scoping reviews, primary studies, opinion/editorial manuscripts, letters to the editor, and reviews of mobile health interventions repositories (i.e. apps stores). Studies in which some of these subjects has participated will also be excluded: a) Adults with some other mental health problem; b) Healthy adults without mental health problems; c) Adults receiving emergency/crisis psychiatric care; d) Interventions that lack a synchronic component (that lack real time information exchange using technologies).

### Information sources

Included databases will be MEDLINE (Ovid), EBM Reviews (Ovid), PsycINFO (Ovid), EMBASE (Elsevier), SCOPUS, CINAHL Complete (EBSCOhost), and Web of Science databases, including Science Citation Index Expanded, Social Sciences Citation Index and Conference Proceedings Citation Index (Clarivate Analytics). Articles published in the last five years (after January 1st, 2015) will be included and no language restrictions will be imposed. We will include systematic reviews that report a review of literature using a research strategy in at least two different databases. Later, all references of included studies will be reviewed, and they will be evaluated looking for any additional systematic review that meets the inclusion criteria.

### Search strategy

Main search terms to be used are "telemedicine" AND "mental health, anxiety, depression or stress" AND “systematic reviews”. The full search strategy for each database is available in the *Extended data* section ^18^.

### Study records

*Data management:* The results of the database search will be managed using the Rayyan QCRI free online application to manage the references (eliminate duplicates, and review titles and summaries) ^20^. Full-text review and data extraction will be done using an Excel template ( *Extended data* ^18^).

*Selection process:* There will be two distinct selection stages. First, a review of the title and abstract and the second a review of the full text. The reviews will be carried out based on pairs of reviewers, who will divide the total number of records. At each stage, there will be a first part where the reviewers will calibrate the accuracy of their reviews and a second part where the actual review will take place. To complete the calibration part, they will make calibration rounds until the discrepancy is less than or equal to 5% of the assigned records. If the percentage of the discrepancy is exceeded, a new round will be performed. Subsequently pairs of reviewers will divide the total of the records identified, and records will be screened independently and in duplicate. Discrepancies will be discussed within pairs with a third reviewer if needed.

In the review by title and abstract, first the calibration will be done, 50 records were selected from the total of records found as a result of the search strategy. Each pair had to make an independent review by title and abstract, selecting the studies that met the inclusion criteria. In the selection stage itself, the number of total records will be divided among all pairs of reviewers.

In the full-text review, first, a calibration of 10 records that will select in the previous phase will be performed. Only articles selected by title and abstract will review in full text. The articles excluded in the full-text review stage will be listed and a reason for exclusion will be given for each one of them.

*Data collection process:* For each eligible study, data will be extracted independently and in duplicate on pre-designed data extraction forms. In the event of discrepancies, the reviewers will discuss whether the extracted data should be retained and decide to resolve the discrepancy. If the discrepancy remains, a third reviewer will evaluate any unresolved disagreement between the reviewers on the data extraction and will decide on the value extracted that is in dispute.

### Data items

An extraction form will be created for the included systematic reviews. Information will be collected on the author and date of the study, characteristics of the participants, main objective, research questions, inclusion criteria for the systematic review, search date, study selection process, quality assessment (if any), main findings and limitations. Also, the full text of the included article will be extracted along with the tables and supplementary material, to perform the qualitative analysis of the text.

### Outcomes and prioritization

Our study seeks to make a meta-synthesis of the systematic reviews, using a qualitative strategy to synthesize the information and answer our research question. Therefore, we do not look for a specific result such as effectiveness, cost-effectiveness or other similar ones. Instead, we are interested in identifying the full text of all studies that answer our research question, to be able to perform a grounded theory analysis with an emergent approach. Priority will be given in the analysis of those studies with the lowest risk of bias assessed.

### Risk of bias in individual studies

To assess the quality of the included systematic reviews we will use the "A Measurement Tool to Assess Systematic Reviews-2" (AMSTAR 2), which has sixteen domains. Seven of these domains are considered critical: 1) protocol registered before the start of the review, 2) adequacy of the literature search, 3) justification for the exclusion of individual studies, 4) risk of bias of individual studies included in the review, 5) adequacy of meta-analytic methods, 6) consideration of the risk of bias in interpreting the results of the review, and 7) assessment of the presence and likely impact of publication bias ^21^.

AMSTAR-2 classifies the quality of systematic reviews into four different categories: high (none or one non-critical weakness), moderate (more than one non-critical weakness), low (one critical weakness with or without non-critical weaknesses), and very low (more than one critical weakness with or without non-critical weaknesses). The quality assessment of each systematic review will be rated by two researchers independently. In case of difference in the overall quality of the systematic reviews, the AMSTAR-2 criteria will be discussed among the researchers to reach a consensus.

### Data synthesis

The analysis of the data will be carried out using a grounded theory approach with an emergent approach ^22, 23^. The reviewers will follow the three steps established by Thomas and Harden ^24^. First, the extracted data are freely coded. The reviewers read the full texts of the included articles and code each text fragment that provides information to answer the research question. Second, the codified data are organized and grouped based on the descriptive aspects. The reviewers will review the codes generated in the previous step and group them into codes that are like each other and that allow the description of a part of the research question. Third, analytical concepts will generate different groups describing aspects generated in the previous step. The meta-synthesis will be based on interpreting, integrating and, inferring the evaluation elements that would allow a better understanding of the e-health implementation process from all the studies included ^24^. In addition to generate hypotheses supported by the results of the included studies, which will be generated through discussion and consensus among the reviewers ^24^.

When included studies had been selected, they will be ranked based on the AMSTAR-2 score, with the highest quality studies being assessed first. We will assess all included studies, down to the criterion of theoretical saturation. All qualitative analyses will be performed with Atlas.ti v.8 software.

### Confidence in cumulative evidence

It will be evaluated the approach of Confidence in the Evidence from Reviews of Qualitative research (CERQual) which has four components (Methodological Limitations, Relevance, Coherence and Adequacy data), and thus contribute to an overall assessment for each systematic review to determine the level of confidence (high, moderate, low, or very low) and present the overall assessment in a Summary of Qualitative Findings (SoQF) table ^25, 26^.

### Dissemination plan

We will present the systematic review findings and submit the manuscript to a relevant peer-reviewed journal.

## Study status

This systematic review is currently in the first stage (title and abstract screening) in the selection process. The protocol of this systematic review was submitted to PROSPERO registry on 18 ^th^ August, 2020 (CRD42020203811).

## Data availability

### Underlying data

No data are associated with this article.

### Extended data

Open Science Framework: PRISMA-P checklist for ‘Development of a framework for the implementation of electronic interventions in mental health: A protocol for a meta-synthesis of systematic reviews’, https://doi.org/10.17605/OSF.IO/3UH4N ^18^. (Registered on 20 ^th^ October 2020: osf.io/tf4b6.)

This project contains the following extended data:

- Full text protocol
- Full search strategy for MEDLINE, EBM Reviews, PsycINFO, EMBASE, SCOPUS, CINAHL Complete, and Web of Science databases
- Excel template for data extraction

### Reporting guidelines

Open Science Framework: PRISMA-P checklist for ‘Development of a framework for the implementation of electronic interventions in mental health: A protocol for a meta-synthesis of systematic reviews’, https://doi.org/10.17605/OSF.IO/3UH4N ^18^. (Registered on 20 ^th^ October 2020: osf.io/tf4b6.)

Data are available under the terms of the Creative Commons Zero "No rights reserved" data waiver (CC0 1.0 Public domain dedication).
