# Development of a framework for the implementation of synchronous e-mental health: a protocol for a realist synthesis of systematic reviews

**DOI:** 10.12688/f1000research.27150.2

**Published:** 2021-08-03

**Authors:** David Villarreal-Zegarra, Christoper A. Alarcon-Ruiz, G.J. Melendez-Torres, Roberto Torres-Puente, Juan Ambrosio-Melgarejo, Alejandra B. Romero-Cabrera, Alba Navarro-Flores, Leonardo Albitres-Flores, Ana Lindo-Cavero, Jeff Huarcaya-Victoria

**Affiliations:** 1 Universidad César Vallejo, Escuela de Medicina, Trujillo, Peru; 2 Instituto Peruano de Orientación Psicológica, Lima, Peru; 3 Universidad Científica del Sur, Lima, Peru; 4 Peninsula Technology Assessment Group, College of Medicine and Health, University of Exeter, Devon, UK; 5 Facultad de Medicina, Universidad Nacional Federico Villarreal, Lima, Peru; 6 CRONICAS Center of Excellence in Chronic Diseases, Universidad Peruana Cayetano Heredia, Lima, Peru; 7 Facultad de Medicina, Universidad Nacional de Trujillo, Trujillo, Peru; 8 Centro de Investigación en Salud Pública, Facultad de Medicina, Universidad de San Martín de Porres, Lima, Peru; 9 Departamento de Psiquiatría, Hospital Nacional Guillermo Almenara Irigoyen, Lima, Peru

**Keywords:** Telemedicine, Remote Consultation, Online Systems, Internet-Based Intervention, Mental Health, Mental Disorders, Systematic Reviews as Topic, Qualitative Research

## Abstract

**Background: **During the COVID-19 pandemic, it has been necessary to deliver mental health care using technologies (e-mental health). But there have been difficulties in its application. Quantitative systematic reviews such as meta-analysis doesn’t allow us to fully identify and properly describe this subject. Thus, our study has two main objectives: a) "to determine what evidence is available for synchronous e-mental health implementation"; and b) "to develop a framework informed by a realist analysis for the implementation of synchronous e-mental health".

**Methods: **We will search MEDLINE, EBM Reviews, PsycINFO, EMBASE, SCOPUS, CINAHL Complete, and Web of Science databases from 1st January 2015 to September 2020, with no language restriction. A systematic review with a narrative description and a realist synthesis will be conducted. Primary studies relating to adults with common mental health problems using any type of mobile mental health intervention that includes a synchronic component and communication with a mental health professional will be included. For the analysis, we will make a realist synthesis of the systematic reviews, using a grounded theory approach with an emergent approach to synthesize the information, prioritizing the systematic reviews with a lower risk of bias in the AMSTAR-2 tool. The realist synthesis will be based on the interpretation, integration, and inference of the evaluated elements and the generation of hypotheses to better understand the implementation process of synchronous e-mental health. Finally, we will present the overall assessment in a Summary of Qualitative Findings table.

**Conclusion: **Our results will allow a better understanding of the facilitator and limitations in implementing e-mental health.

## Introduction

In the wake of the COVID-19 pandemic and after the actions taken by governments (such as social isolation), many mental health problems have increased in patients with COVID-19, patients with psychiatric symptoms, health personnel, and the general population ^1, 2^. As a result, greater interest has been taken in addressing mental health issues during the pandemic ^2, 3^. Studies have identified anxiety disorders, depression, post-traumatic stress disorder, and stress as the most frequent health problems ^2– 4^. As a result, it has been necessary to deliver mental health care through technology (e-mental health) such as internet and mobile devices ^5, 6^ These kind of health care have been very well received and have served to complement or improve the effectiveness of treatments for various chronic diseases, reducing gaps in access to care and providing specialized care in inaccessible places ^7^ and it shows great promise in the care of mental health problems ^8– 10^, highlighting the possibilities of care in health systems where resources are limited ^11^.

With the undeniable contribution of e-mental health, it has been important to document the aspects related to its application because, despite its effectiveness, it is known that there have been difficulties in its application ^12^. Aspects such as adaptability, cost, complexity, external policies and incentives, compatibility or general fit between the e-mental health intervention and the organization ^13^ are important to consider to be clear about how and what works in synchronous and electronic interventions in mental health (synchronous e-mental health), and considering its complexity as well. To address this, systematic reviews and meta-analyses have given us knowledge about the effectiveness of e-mental health ^14, 15^. However, qualitative studies are necessary because of their methodology (approach to answer questions about experience, meaning, and perspective) which is useful to address issues of implementation ^16^. Thus, our study has two main objectives: a) "to determine what evidence is available for synchronous e-mental health implementation"; and b) "to develop a framework informed by a realist analysis for the implementation of synchronous e-mental health".

## Methods

### Research question

This protocol adheres to the Preferred Reporting Items for Systematic Reviews and Meta-Analyses Protocols (PRISMA-P) guidelines ^17^ A completed PRISMA-P checklist can be found in the *Reporting guidelines* section ^18^. The SPIDER framework was used to develop the review question, which is based on describing the Sample (S), Phenomenon of Interest (PI), Design (D), Evaluation (E), and Research type (R) ^16^:

• *Sample:* Adults with depression (or major depressive disorder), anxiety (or generalized anxiety disorder), stress (or trauma-related disorders), and/or general mental health problems (unspecified). Participants may be diagnosed through clinical interviews or categorized based on screening assessments (self-reported scales).
• *Phenomenon of interest:* Any type of e-mental health intervention that includes a synchronous component, communication with a mental health professional (psychiatrist, psychologist, etc.) or a health professional trained in mental health. These interventions include, among others, remote consultation, interactive application, video chats, calls, etc.
• *Design:* Systematic review.
• *Evaluation:* All types of outcomes of interest assessed by implementation, economic, qualitative, quantitative, and other studies will be included. For example, a) Health effectiveness outcomes: Depression, anxiety and/or stress symptoms, adherence to treatment, etc; b) Patient outcomes: Quality of life, satisfaction, etc; c) Economic outcomes; d) Damage or adverse effects. Our study seeks to perform a realist synthesis of the evidence, so we focused on different outcomes to use them as input that allows us to evaluate the implementation of synchronous e-mental health. Therefore, we do not perform a quantitative synthesis in any case (i.e., a meta-analysis of effectiveness).
• *Research type:* Quantitative studies, qualitative studies, mixed methods.

### Eligibility criteria

*Inclusion criteria:* Systematic reviews using at least two databases that synthesized, assessed the quality, and presented sufficient detail of the primary included studies; and reports on inclusion/exclusion criteria ^19^. Systematic reviews that included primary studies as a unit of analysis focused on a research question. Systematic reviews published in the last five years (since January 1, 2015) without language restrictions. We include this time frame to include only the latest systematic reviews, since in the field of e-health the launch of new technologies makes scientific development dynamic. Reviews must include primary studies relating to adults with common mental health problems: a) Adults with depression (or major depressive disorder), anxiety (or generalized anxiety disorder), stress (or trauma-related disorders) and/or general mental health problems (unspecified); b) Adults attending an outpatient mental health consultation. Reviews must include at least 90% of their primary studies that assess synchronous e-mental health or presenting its results independently only for synchronous e-mental health.

*Exclusion criteria:* Narrative reviews, scoping reviews, primary studies, opinion/editorial manuscripts, letters to the editor, and reviews of mobile health interventions repositories (i.e., apps stores). Studies in which some of these subjects has participated will also be excluded: a) Adults with some other mental health problem; b) Healthy adults without mental health problems; c) Adults receiving emergency/crisis psychiatric care; d) Interventions that lack a synchronous component (real time information exchange between the user and mental health professional using technologies) or are not sufficiently clear that had a synchronous component.

### Information sources

Included databases will be MEDLINE, EBM Reviews, PsycINFO (these three through Ovid), EMBASE (Elsevier), SCOPUS, CINAHL Complete (EBSCOhost), and Web of Science databases, including Science Citation Index Expanded, Social Sciences Citation Index and Conference Proceedings Citation Index (Clarivate Analytics). Articles published in the last five years (after January 1st, 2015) will be included and no language restrictions will be imposed. We will include systematic reviews that report a review of literature using a research strategy in at least two different databases. Later, all references of included studies will be reviewed, and they will be evaluated looking for any additional systematic review that meets the inclusion criteria.

### Search strategy

Main search terms to be used are "telemedicine" AND "mental health, anxiety, depression or stress" AND “systematic reviews”. The full search strategy for each database is available in the *Extended data* section ( https://osf.io/c4jua/) ^18^.

### Study records

*Data management:* The results of the database search will be managed using the Rayyan QCRI free online application to manage the references (eliminate duplicates, and review titles and summaries) ^20^. Full-text review and data extraction will be done using an Excel template ( https://osf.io/c4jua/ ^18 ^).

*Selection process:* There will be two distinct selection stages. First, a review of the title and abstract and the second a review of the full text. The reviews will be carried out based on pairs of reviewers, who will divide the total number of records. At each stage, there will be a first part where the reviewers will calibrate the accuracy of their reviews and a second part where the actual review will take place. To complete the calibration part, they will make calibration rounds until the discrepancy is less than or equal to 5% of the assigned records. If the percentage of the discrepancy is exceeded, a new round will be performed. Subsequently pairs of reviewers will divide the total of the records identified, and records will be screened independently and in duplicate. Discrepancies will be discussed within pairs with a third reviewer if needed.

In the review by title and abstract, calibration will be done first, 50 records were selected from the total number of records found because of the search strategy. In the full-text review, calibration will first be performed on 10 records that will be selected in the previous stage. The articles excluded in the full-text review stage will be listed and a reason for exclusion will be given for each of them.

*Data collection process:* For each eligible study, data will be extracted independently and in duplicate on pre-designed data extraction forms. In the event of discrepancies, the reviewers will discuss whether the extracted data should be retained and decide to resolve the discrepancy. If the discrepancy remains, a third reviewer will evaluate any unresolved disagreement between the reviewers on the data extraction and will decide on the value extracted that is in dispute.

### Data items

An extraction form was created in an Excel template for the included systematic reviews ( https://osf.io/7esgz/). Information will be collected on the author and date of the study, characteristics of the participants, main objective, research questions, inclusion criteria for the systematic review, search date, study selection process, quality assessment (if any), main findings and limitations. Also, the full text of the included article will be extracted along with the tables and supplementary material, to perform the qualitative analysis of the text.

### Outcomes and prioritization

Our study aims to develop a framework informed by a realist analysis for the implementation of synchronous e-mental health, using a qualitative strategy to synthesize the information and answer our research question. Therefore, we do not look for specific results such as effectiveness, cost-effectiveness, or other similar ones. Instead, we are interested in identifying the full text of all the studies that answer our research question, so that we can make a realistic synthesis using a grounded theory model with an emergent approach. The develop a framework informed by a realist analysis will be based on the interpretation, integration, and inference of the evaluated elements and the generation of hypotheses to better understand the studied phenomenon (implementation of synchronous e-mental health) ^21, 22^. Priority will be given in the analysis of those studies with the lowest risk of bias assessed. If possible, a realist synthesis will be performed only with those systematic reviews with high quality, to assess whether the results change by selecting only those studies with high quality (low risk of bias).

### Risk of bias in individual studies

To assess the quality of the included systematic reviews we will use the "A Measurement Tool to Assess Systematic Reviews-2" (AMSTAR 2), which has sixteen domains. Seven of these domains are considered critical: 1) protocol registered before the start of the review, 2) adequacy of the literature search, 3) justification for the exclusion of individual studies, 4) risk of bias of individual studies included in the review, 5) adequacy of meta-analytic methods, 6) consideration of the risk of bias in interpreting the results of the review, and 7) assessment of the presence and likely impact of publication bias ^23^.

AMSTAR-2 classifies the quality of systematic reviews into four different categories: high (none or one non-critical weakness), moderate (more than one non-critical weakness), low (one critical weakness with or without non-critical weaknesses), and very low (more than one critical weakness with or without non-critical weaknesses). The quality assessment of each systematic review will be rated by two researchers independently. In case of difference in the overall quality of the systematic reviews, the AMSTAR-2 criteria will be discussed among the researchers to reach a consensus.

### Data synthesis

The develop a framework informed by a realist analysis for the implementation of synchronous e-mental health will be carried out using a grounded theory approach with an emergent approach ^24^. The realist synthesis will be based on interpreting, integrating and, inferring the evaluation elements that would allow a better understanding of the synchronous e-mental health implementation process from all the studies included ^24^. To answer the question "what makes the implementation of these interventions work", hypotheses supported by the results of the included studies will be developed and generated through discussion and consensus among researchers ^24^. Two reviewers will follow the three steps established by Thomas and Harden for qualitative syntheses will be followed ^25, 26^. First, the extracted data are freely coded. The reviewers read the full texts of the included articles and code each text fragment that provides information to answer the research question. Second, the codified data are organized and grouped based on the descriptive aspects. The reviewers will review the codes generated in the previous step and group them into codes that are like each other and that allow the description of a part of the research question. Thirdly, the analytical concepts generated in the previous step will be grouped in a way that they are related to each other. The elements that are related to each other will be assumed to be part of a hypothesis that would help to answer the research aim. The sum of all these hypotheses will be referred to as a framework which will attempt to explain the results using a context-linked causality approach represented as context + mechanism = outcome ^26^.

When included studies had been selected, they will be ranked based on the AMSTAR-2 score, with the highest quality studies being assessed first. We will assess all included studies, down to the criterion of theoretical saturation. All qualitative analyses will be performed with NVIVO v.1 software.

### Confidence in cumulative evidence

It will be evaluated the approach of Confidence in the Evidence from Reviews of Qualitative research (CERQual) which has four components (Methodological Limitations, Relevance, Coherence and Adequacy data), will be evaluated to contribute to an overall assessment for each hypothesis resulting from the realist synthesis to determine the level of confidence (high, moderate, low, or very low) and present the overall assessment in a Summary of Qualitative Findings (SoQF) table ^27, 28^.

### Dissemination plan

We will present the systematic review findings and submit the manuscript to a relevant peer-reviewed journal.

## Study status

This systematic review is currently in the data analysis process. The protocol of this systematic review was submitted to PROSPERO registry on 18 ^th^ August, 2020 (CRD42020203811).

## Data availability

### Underlying data

No data are associated with this article.

### Extended data

Open Science Framework: PRISMA-P checklist for ‘Development of a framework for the implementation of electronic interventions in mental health: A protocol for a meta-synthesis of systematic reviews’, https://doi.org/10.17605/OSF.IO/3UH4N ^18^. (Registered on 20 ^th^ October 2020: osf.io/tf4b6.)

This project contains the following extended data:

- Full text protocol
- Full search strategy for MEDLINE, EBM Reviews, PsycINFO, EMBASE, SCOPUS, CINAHL Complete, and Web of Science databases
- Excel template for data extraction

### Reporting guidelines

Open Science Framework: PRISMA-P checklist for ‘Development of a framework for the implementation of electronic interventions in mental health: A protocol for a meta-synthesis of systematic reviews’, https://doi.org/10.17605/OSF.IO/3UH4N ^18^. (Registered on 20 ^th^ October 2020: osf.io/tf4b6.)

Data are available under the terms of the Creative Commons Zero "No rights reserved" data waiver (CC0 1.0 Public domain dedication).
