# Supplementary material for: Development of a framework for the implementation of synchronous e-mental health: a protocol for a realist synthesis of systematic reviews
Source: F1000Res. 2021 Aug 3;9:1282. Originally published 2020 Oct 29. [Version 2] doi: 10.12688/f1000research.27150.2 (PMC8424460; doi:10.12688/f1000research.27150.2)
Supplement: Peer review history

# Peer review: Reviewer response for version 2

**DOI:** 10.5256/f1000research.58449.r91140

**Published:** 2021-09-07

**Authors:** Roger Hilfiker

**Affiliations:** 1 Valais-Wallis School of Health Sciences, HES-SO, University of Applied Sciences and Arts Western Switzerland, Valais, Switzerland

Thank you very much for the changes made.

Is the study design appropriate for the research question?

Yes

Is the rationale for, and objectives of, the study clearly described?

Yes

Are sufficient details of the methods provided to allow replication by others?

Yes

Are the datasets clearly presented in a useable and accessible format?

Yes

Reviewer Expertise:

Systematic Reviews

I confirm that I have read this submission and believe that I have an appropriate level of expertise to confirm that it is of an acceptable scientific standard.

# Peer review: Reviewer response for version 2

**DOI:** 10.5256/f1000research.58449.r91141

**Published:** 2021-08-17

**Authors:** Amy Grove

**Affiliations:** 1 Health Technology Assessment and Implementation Science, Warwick Medical School, University of Warwick, Coventry, UK

I am satisfied with the revisions.

Is the study design appropriate for the research question?

Yes

Is the rationale for, and objectives of, the study clearly described?

Yes

Are sufficient details of the methods provided to allow replication by others?

Yes

Are the datasets clearly presented in a useable and accessible format?

Yes

Reviewer Expertise:

HTA and implementation science

I confirm that I have read this submission and believe that I have an appropriate level of expertise to confirm that it is of an acceptable scientific standard.

# Peer review: Reviewer response for version 1

**DOI:** 10.5256/f1000research.29992.r85471

**Published:** 2021-06-18

**Authors:** Roger Hilfiker

**Affiliations:** 1 Valais-Wallis School of Health Sciences, HES-SO, University of Applied Sciences and Arts Western Switzerland, Valais, Switzerland

The manuscript reports on a protocol for a systematic review with a meta-synthesis of systematic reviews. 

 The authors describe a qualitative approach for the analysis of the data with three steps, i.e. coding of each text fragment that provides answers to the research questions, organizing and grouping the codified data, and grouping the aspects. 

 I have some specific comments: 

 Research question: Please clarify how you will use the data to answer the question on "what  makes the application of any of these interventions work". 

 Data synthesis: The sentence "In addition to generate hypotheses supported by the results of the included studies, which will be generated through discussion and consensus among the reviewers" is not clear to me. 

 The authors described the extraction of quantitative data on effectiveness. Please clarify whether these quantitative data are analyzed with the qualitative method or whether quantitative methods are used. 

 Please describe how the research question on the "what makes the application work" will be answered. 

 Confidence in cumulative evidence: Wouldn't the research question "what makes the application of any of these interventions work" require a different evaluation of the confidence of the evidence, for example an approach such as GRADE?

Is the study design appropriate for the research question?

Yes

Is the rationale for, and objectives of, the study clearly described?

Yes

Are sufficient details of the methods provided to allow replication by others?

Yes

Are the datasets clearly presented in a useable and accessible format?

Yes

Reviewer Expertise:

Systematic Reviews

I confirm that I have read this submission and believe that I have an appropriate level of expertise to confirm that it is of an acceptable scientific standard, however I have significant reservations, as outlined above.

# Response

**Published:** 2021-07-06

**Authors:** Christoper A. Alarcon-Ruiz

**Affiliations:** Universidad Científica del Sur, Peru

1. Research question: Please clarify how you will use the data to answer the question on "what makes the application of any of these interventions work". 

 Reply: It was specified in the subsection of Data synthesis

 “The develop a framework informed by a realist analysis for the implementation of synchronous e-mental health will be carried out using a grounded theory approach with an emergent approach (23). The realist synthesis will be based on interpreting, integrating and, inferring the evaluation elements that would allow a better understanding of the synchronous e-mental health implementation process from all the studies included (24). To answer the question "what makes the implementation of these interventions work", hypotheses supported by the results of the included studies will be developed and generated through discussion and consensus among researchers (24). Two reviewers will follow the three steps established by Thomas and Harden for qualitative syntheses will be followed (25). First, the extracted data are freely coded. The reviewers read the full-texts of the included articles and code each text fragment that provides information to answer the research question. Second, the codified data are organized and grouped based on the descriptive aspects. The reviewers will review the codes generated in the previous step and group them into codes that are like each other and that allow the description of a part of the research question. Thirdly, the analytical concepts generated in the previous step will be grouped in a way that they are related to each other. The elements that are related to each other will be assumed to be part of a hypothesis that would help to answer the research aim. The sum of all these hypotheses will be referred to as a framework which will attempt to explain the results using a context-linked causality approach represented as context + mechanism = outcome (26).”

 2. Data synthesis: The sentence "In addition to generate hypotheses supported by the results of the included studies, which will be generated through discussion and consensus among the reviewers" is not clear to me. 

 Reply: We agree with the reviewer, this has been modified in the previous comment. 

 3. The authors described the extraction of quantitative data on effectiveness. Please clarify whether these quantitative data are analyzed with the qualitative method or whether quantitative methods are used. 

 Reply: The collection of quantitative data was eliminated, as specified in the method section: 

 “Our study seeks to perform a realistic synthesis of the evidence, so we focused on different outcomes to use them as input. Therefore, we do not perform a quantitative synthesis in any case (i.e., a meta-analysis of effectiveness).”

 4. Please describe how the research question on the "what makes the application work" will be answered.

 Reply: A better description of the research objective was made based on the first reviewer.

 “Thus, our study has two main objectives: a) "to determine what evidence is available for synchronous e-mental health implementation"; and b) "to develop a framework informed by a realist analysis for the implementation of synchronous e-mental health".”

 The process of how to achieve these objectives was answered in the first comment.

 5. Confidence in cumulative evidence: Wouldn't the research question "what makes the application of any of these interventions work" require a different evaluation of the confidence of the evidence, for example an approach such as GRADE?

 Reply: we will evaluate each key finding using CERQual to assess the methodological limitations, coherence, adequacy, and relevance of findings.

 “It will be evaluated the approach of Confidence in the Evidence from Reviews of Qualitative research (CERQual) which has four components (Methodological Limitations, Relevance, Coherence and Adequacy data), will be evaluated to contribute to an overall assessment for each hypothesis resulting from the realist synthesis to determine the level of confidence (high, moderate, low, or very low) and present the overall assessment in a Summary of Qualitative Findings (SoQF) table (27, 28).”

# Peer review: Reviewer response for version 1

**DOI:** 10.5256/f1000research.29992.r78252

**Published:** 2021-02-11

**Authors:** Amy Grove

**Affiliations:** 1 Health Technology Assessment and Implementation Science, Warwick Medical School, University of Warwick, Coventry, UK

**Abstract:**

- " *Some quantitative systematic*" can you indicate how many rather than using the generic some.
- Would the research question be better phrased as 2 questions? or a primary and secondary question? i) how question?; ii) what makes question? 
- Please define what approach you will use *"We will follow a qualitative method approach".*
- Are *"incorporate technologies", "implementation of electronic care", "mobile mental health intervention that includes a synchronic component and communication with a mental health professional", "e-health implementation process" and, "synchronic component (that lack real time information exchange using technologies)."* all the same thing? I would suggest you select one terminology and use it consistently. 

**Introduction: **

- Please remove *"To deal with these mental health problems".* This is not appropriate terminology.
- You talk about *"These technologies",* can you provide examples?
- You say *"(centered on quantitative studies) does not allow to identify".* Describe what it does not allow; what is missing from existing reviews?

**Methods: **

- The heading, " *SPIDER question", *needs to be replaced with *"research question"*. The acronym may not be understood by all. 
- Regarding the sample - do people have to have a certified diagnosis of the condition? What about undiagnosed groups? 

**Evaluation:**

- Are you assessing effectiveness? or just implementation of a technology that has shown to be effective elsewhere?
- If you are following a *"qualitative method approach"* why are you including outcomes of *"all kinds of statistical measures of the effects"*? What will you do with this information?
- How do you define *"conducted an adequate search"?* If you are using a criteria then please list it.
- Please provided references for *"only the last updated systematic reviews".*
- The study selection process is overly detailed and repetitive. 
- You say *"An extraction form will be created for the included systematic reviews.",* but above you imply it is already done and included in *"an Excel template (Extended data)"*.
- *"Therefore, we do not look for a specific result such as effectiveness, cost-effectiveness or other similar ones."* Then why are you searching for data? 
- *"perform a grounded theory analysis with an emergent approach"* - you said earlier *"using an emergent grounded theory approach",* what is the difference? If no difference, then please use consistent terms. 
- *"Priority will be given in the analysis of those studies with the lowest risk of bias assessed." - *Explain why this is the case
- In data synthesis you do not state how many *"reviewers will follow the three steps".*
- In the abstract you say you will *"develop a theoretical framework"* but you do not give detail of how this will be done in the manuscript. 
- Please also proof read for typographical errors and appropriate use of English. 

Is the study design appropriate for the research question?

Yes

Is the rationale for, and objectives of, the study clearly described?

Yes

Are sufficient details of the methods provided to allow replication by others?

Yes

Are the datasets clearly presented in a useable and accessible format?

Yes

Reviewer Expertise:

HTA and implementation science

I confirm that I have read this submission and believe that I have an appropriate level of expertise to confirm that it is of an acceptable scientific standard, however I have significant reservations, as outlined above.

# Response

**Published:** 2021-07-06

**Authors:** Christoper A. Alarcon-Ruiz

**Affiliations:** Universidad Científica del Sur, Peru

**Abstract:**

 1. "Some quantitative systematic" can you indicate how many rather than using the generic some.

 Reply: We specified this concern. Abstract: “Quantitative systematic reviews such as meta-analysis” 

 2. Would the research question be better phrased as 2 questions? or a primary and secondary question? i) how question?; ii) what makes question?

 Reply: It was modified in the abstract:

 “Thus, our study has two main objectives: a) "to determine what evidence is available for synchronous e-mental health implementation"; and b) "to develop a framework informed by a realistic analysis for the implementation of synchronous e-mental health".”

 3. Please define what approach you will use "We will follow a qualitative method approach".

 Reply: It was modified in the abstract:

 “A systematic review with narrative description and a realist synthesis will be conducted. Primary studies related to adults with common mental health problems using any type of mobile mental health intervention that includes a synchronous component and communication with a mental health professional will be included. For the analysis, we will make a realistic synthesis of the systematic reviews, using a grounded theory approach with an emergent approach to synthesize the information, prioritizing the systematic reviews with a lower risk of bias in the AMSTAR-2 tool. The realist synthesis will be based on the interpretation, integration, and inference of the evaluated elements and the generation of hypotheses to better understand the implementation process of synchronic e-mental health.”

 4. Are "incorporate technologies", "implementation of electronic care", "mobile mental health intervention that includes a synchronic component and communication with a mental health professional", "e-health implementation process" and, "synchronic component (that lack real-time information exchange using technologies)." all the same thing? I would suggest you select one terminology and use it consistently.

 Reply: We’ll use the term “e-mental health” to refer to mental health care delivery through technology. And we’ll use “synchronous e-mental health” to refer to the delivery of mental health care through technology in real-time.

**Introduction:**

 5. Please remove "To deal with these mental health problems". This is not appropriate terminology.

 Reply: We changed it for “As a result, it has been...” 

 6. You talk about "These technologies", can you provide examples?

 Reply: We specified some examples: “such as internet and mobile devices ” 

 7. You say "(centered on quantitative studies) does not allow to identify". Describe what it does not allow; what is missing from existing reviews?

 Reply: Does not provide information about experience, meaning, and perspective, which is important when addressing implementation issues.

 “However, qualitative studies are necessary because of their methodology (approach to answer questions about experience, meaning, and perspective) which is useful to address issues of implementation.”

**Methods:**

 8. The heading, "SPIDER question", needs to be replaced with "research question". The acronym may not be understood by all.

 Reply: The heading has been modified.

 9. Regarding the sample - do people have to have a certified diagnosis of the condition? What about undiagnosed groups? 

 Reply: It was modified in the methods:

 “Sample: Adults with depression (or major depressive disorder), anxiety (or generalized anxiety disorder), stress (or trauma-related disorders), and/or general mental health problems (unspecified). Participants may be diagnosed through clinical interviews or categorized based on screening assessments (self-reported scales).”

**Evaluation:**

 10. Are you assessing effectiveness? or just implementation of a technology that has shown to be effective elsewhere?

 Reply: It was modified in the method:

 “All types of outcomes of interest assessed by implementation, economic, qualitative, quantitative, and other studies will be included. For example, a) Health effectiveness outcomes: Depression, anxiety and/or stress symptoms, adherence to treatment, etc.; b) Patient outcomes: Quality of life, satisfaction, etc.; c) Economic outcomes; d) Damage or adverse effects. Our study seeks to perform a realist synthesis of the evidence, so we focused on different outcomes to use them as input that allow s us to evaluate the implementation of synchronous e-mental health. Therefore, we do not perform a quantitative synthesis in any case (i.e., a meta-analysis of effectiveness).”

 11. If you are following a "qualitative method approach" why are you including outcomes of "all kinds of statistical measures of the effects"? What will you do with this information?

 Reply: We agree with the reviewer. The quantitative information was eliminated. It was modified in the method:

 “Our study seeks to perform a realist synthesis of the evidence, so we focused on different outcomes to use them as input. Therefore, we do not perform a quantitative synthesis in any case (i.e., a meta-analysis of effectiveness).”

 12. How do you define "conducted an adequate search"? If you are using criteria then please list them.

 Reply: We agree with your observation. We have specified and listed the criteria we consider to include a systematic review “Systematic reviews using at least two databases that synthesized, assessed the quality, and presented sufficient detail of the primary included studies; and reports on inclusion/exclusion criteria”

 13. Please provided references for "only the last updated systematic reviews".

 Reply:  It was specified in the text:

 “We include this time frame to include only the latest systematic reviews since in the field of e-health, the launch of new technologies makes scientific development dynamic. “

 14. The study selection process is overly detailed and repetitive. 

 Reply: We synthesized the study selection:

 “In the review by title and abstract, calibration will be done first, 50 records were selected from the total number of records found as a result of the search strategy. In the full-text review, calibration will first be performed on 10 records that will be selected in the previous stage. The articles excluded in the full-text review stage will be listed, and a reason for exclusion will be given for each of them.”

 15. You say, "An extraction form will be created for the included systematic reviews.", but above, you imply it is already done and included in "an Excel template (Extended data)".

 Reply: It was modified in the subsection of data items:

 “An extraction form was created in an excel template for the included systematic reviews (https://osf.io/7esgz/).”

 16. "Therefore, we do not look for a specific result such as effectiveness, cost-effectiveness or other similar ones." Then why are you searching for data? 

 Reply: It was modified in the method:

 “Our study seeks to perform a realist synthesis of the evidence, so we focused on different outcomes to use them as input. Therefore, we do not perform a quantitative synthesis in any case (i.e., a meta-analysis of effectiveness).”

 The section where we state that we will collect specific quantitative data, such as effect size, has been removed. Studies with efficacy, cost-effectiveness, or other similar outcomes are included because they will help answer our question.

 17. "perform a grounded theory analysis with an emergent approach" - you said earlier, "using an emergent grounded theory approach", what is the difference? If no difference, then please use consistent terms. 

 Reply: It was specified:

 “Our study aims to conduct a systematic review of systematic reviews and a realistic synthesis of systematic reviews, using a qualitative strategy to synthesize the information and answer our research question. Therefore, we do not look for specific results such as effectiveness, cost-effectiveness, or other similar ones. Instead, we are interested in identifying the full text of all the studies that answer our research question so that we can make a realistic synthesis using a grounded theory model with an emergent approach. The realistic synthesis will be based on the interpretation, integration, and inference of the evaluated elements and the generation of hypotheses to better understand the studied phenomenon (implementation of synchronous e-mental health) (20, 21).” 

 18. "Priority will be given in the analysis of those studies with the lowest risk of bias assessed." - Explain why this is the case

 Reply: It was specified:

 “Priority will be given in the analysis of those studies with the lowest risk of bias assessed. If possible, a realist synthesis will be performed only with those systematic reviews with high quality, to assess whether the results change by selecting only those studies with high quality (low risk of bias).”

 19. In data synthesis, you do not state how many "reviewers will follow the three steps".

 Reply:  We agree with your observation. We have specified the number of reviewers involved in this process. “Two reviewers will follow the three steps established by Thomas and Harden for qualitative syntheses will be followed.”

 20. In the abstract, you say you will "develop a theoretical framework" but you do not give detail of how this will be done in the manuscript.

 Reply: The authors have identified that the meta-synthesis analysis method was not the most appropriate for the review, as the description is more in line with a realistic synthesis analysis. It was specified in the section on data synthesis.

 “The develop a framework informed by a realist analysis for the implementation of synchronous e-mental health will be carried out using a grounded theory approach with an emergent approach (23). The realist synthesis will be based on interpreting, integrating and, inferring the evaluation elements that would allow a better understanding of the synchronic e-mental health implementation process from all the studies included (24). To answer the question "what makes the implementation of these interventions work", hypotheses supported by the results of the included studies will be developed and generated through discussion and consensus among researchers (24). Two reviewers will follow the three steps established by Thomas and Harden for qualitative syntheses will be followed (25). First, the extracted data are freely coded. The reviewers read the full texts of the included articles and code each text fragment that provides information to answer the research question. Second, the codified data are organized and grouped based on the descriptive aspects. The reviewers will review the codes generated in the previous step and group them into codes that are like each other and that allow the description of a part of the research question. Thirdly, the analytical concepts generated in the previous step will be grouped in a way that they are related to each other. The elements that are related to each other will be assumed to be part of a hypothesis that would help to answer the research aim. The sum of all these hypotheses will be referred to as a framework that will attempt to explain the results using a context-linked causality approach represented as context + mechanism = outcome (26). ”

 21. Please also proofread for typographical errors and appropriate use of English. 

 Reply: We accept your observation. We have reviewed and corrected typographical errors and the adequate use of English.
